# Site‐Specific Immobilization of the Peptidoglycan Synthase PBP1B on a Surface Plasmon Resonance Chip Surface

**DOI:** 10.1002/cbic.201600461

**Published:** 2016-11-07

**Authors:** Inge L. van't Veer, Nadia O. L. Leloup, Alexander J. F. Egan, Bert J. C. Janssen, Nathaniel I. Martin, Waldemar Vollmer, Eefjan Breukink

**Affiliations:** ^1^ Department of Membrane Biochemistry and BiophysicsUtrecht UniversityPadualaan 83584 CHUtrechtThe Netherlands; ^2^ Crystal and Structural ChemistryUtrecht UniversityPadualaan 83584 CHUtrechtThe Netherlands; ^3^ The Centre for Bacterial Cell BiologyNewcastle UniversityRichardson RoadNE2 4AX, Newcastle upon TyneUK; ^4^ Department of Chemical Biology and Drug DiscoveryUtrecht Institute for Pharmaceutical SciencesUtrecht UniversityUniversiteitsweg 993584 CGUtrechtThe Netherlands

**Keywords:** click chemistry, PBP, protein modifications, site specific immobilization, surface plasmon resonance

## Abstract

Surface plasmon resonance (SPR) is one of the most powerful label‐free methods to determine the kinetic parameters of molecular interactions in real time and in a highly sensitive way. Penicillin‐binding proteins (PBPs) are peptidoglycan synthesis enzymes present in most bacteria. Established protocols to analyze interactions of PBPs by SPR involve immobilization to an ampicillin‐coated chip surface (a β‐lactam antibiotic mimicking its substrate), thereby forming a covalent complex with the PBPs transpeptidase (TP) active site. However, PBP interactions measured with a substrate‐bound TP domain potentially affect interactions near the TPase active site. Furthermore, in vivo PBPs are anchored in the inner membrane by an N‐terminal transmembrane helix, and hence immobilization at the C‐terminal TPase domain gives an orientation contrary to the in vivo situation. We designed a new procedure: immobilization of PBP by copper‐free click chemistry at an azide incorporated in the N terminus. In a proof‐of‐principle study, we immobilized *Escherichia coli* PBP1B on an SPR chip surface and used this for the analysis of the well‐characterized interaction of PBP1B with LpoB. The site‐specific incorporation of the azide affords control over protein orientation, thereby resulting in a homogeneous immobilization on the chip surface. This method can be used to study topology‐dependent interactions of any (membrane) protein.

## Introduction

Surface plasmon resonance (SPR) is a powerful technique for the kinetic characterization of biomolecular interactions. This technique requires the immobilization of one part of an interacting pair to a sensor‐chip surface, over which the second part is passed in solution. Binding of the soluble analyte to the immobilized ligand generates an SPR sensorgram, which is a plot of arbitrary response or resonance units against time. The resonance units result from the change in refractive index at the chip surface upon analyte binding, as measured by sensitive optical apparatus. The generated data can be used to calculate the kinetic parameters of an interaction, such as association and dissociation rate constants and hence affinity, as well as the equilibrium constant of the interaction.^1^, ^2^, ^3^ Common methods for protein immobilization involve covalent coupling on an SPR chip surface to naturally occurring amine or thiol groups within the protein. Immobilization at amines occurs after reaction with N‐hydroxysuccinimide (NHS) esters coupled to the chip surface during manufacture.^4^ For thiol coupling, reactive disulfide or maleimide groups can be introduced on the chip surface. Other functional groups, such as aldehydes, can also be chemically introduced in the protein to allow specific coupling to the chip surface;^5^ affinity fusion tags, such as glutathione S‐transferase, maltose binding protein, poly‐histidine tags in combination with glutathione, amylose and nickel‐NTA‐functionalized surfaces have also been used.^1^, ^3^ SPR has been used to study numerous interactions in biological systems, including those of penicillin‐binding proteins (PBPs).

PBPs are peptidoglycan (PG) synthesis enzymes responsible for the final steps in the production of the major component of the bacterial cell wall.^6^ PG is a mesh‐like heteropolymer composed of glycan strands interconnected by short peptides, and is synthesized at the outer leaflet of the cytoplasmic membrane. It is synthesized from lipid II by two enzymatic reactions: polymerization of glycan strands by glycosyltransferase (GTase) reactions, and cross‐linkage of peptides by transpeptidase (TPase) reactions.^7^, ^8^, ^9^ PBPs form a family of enzymes with members capable of either TPase activity or both GTase and TPase; they are so named because they readily form covalent complexes with penicillin and other β‐lactam antibiotics at their TPase domains.^10^, ^11^, ^12^ All PG synthases are anchored to the cytoplasmic membrane by a single transmembrane helix, with the catalytic site on the outside. *Escherichia coli* is the best‐studied model organism for the interactions of PG enzymes. PBP1B, a major *E. coli* PG synthase, has both GTase and TPase activities.^13^, ^14^, ^15^ Several interactions of PBP1B have been characterized by SPR, including with other PG synthesis enzymes (PBP3, MltA‐MipA) and regulatory proteins (LpoB, CpoB, FtsN).^16^, ^17^, ^18^, ^19^, ^20^ SPR was also used to demonstrate dimerization of PBP1B.^21^ In all these examples, PBP1B (or PBP3) was immobilized onto the chip surface at its TPase domain. This was achieved by coupling ampicillin to the chip surface and subsequently applying the PBP, thereby resulting in a covalent interaction of ampicillin with the active site of the TPase domain of the PBP.

**Figure 1:**
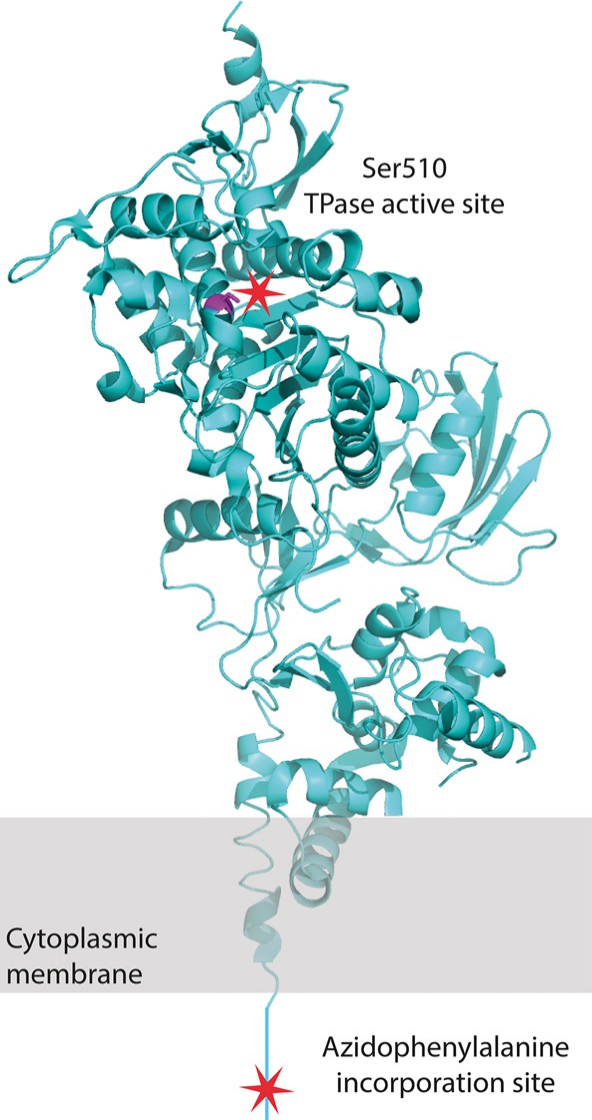
Crystal structure of PBP1B (PDB ID: 3FWL)^34^ showing the previously used immobilization site (serine residue in the active site of the TPase domain) and the site used in our immobilization strategy (cytoplasmic tail).

The oriented coupling of PBPs to the chip surface via ampicillin is suboptimal in some cases. As the TPase active site is occupied in this immobilization strategy, any interaction interfaces proximal to this position can be occluded and/or altered compared to the apo state. Furthermore, in the cell PBPs are anchored in the cytoplasmic membrane by an N‐terminal transmembrane helix, with the majority of the protein oriented outwards and the TPase domain typically furthest from the point of anchoring (Figure 1). Thus, immobilization of a PBP by its TPase domain gives a contrary orientation to that in vivo, thus exposing the GTase domain and membrane anchor and potentially occluding interaction sites or hindering access of analyte molecules.

To address this, we have designed a new immobilization method, based on site‐specific labeling, that can be generally applied to any (membrane) protein. Many different bioorthogonal chemical reactions have been described to site‐specifically label proteins for surface immobilization.^22^, ^23^ We chose to anchor PBP1B to a chip surface by site‐specific incorporation of an azide‐containing unnatural amino acid in the N‐terminal sequence of the protein (Figure 1), followed by covalent attachment to immobilized dibenzylcyclooctyne by copper‐free click chemistry, as this reaction occurs spontaneously under physiological conditions and does not need metal catalysts, which can have undesirable effects on protein activity.^23^ Immobilization by this method yields the correct topological orientation of the protein with an accessible and unaltered TPase domain, thereby allowing characterization of interactions with this domain.

## Results and Discussion

### Confirmation of the presence of the azide in the PBP1B mutants by coupling to a cyclooctyne‐containing fluorescent dye

**Figure 2:**
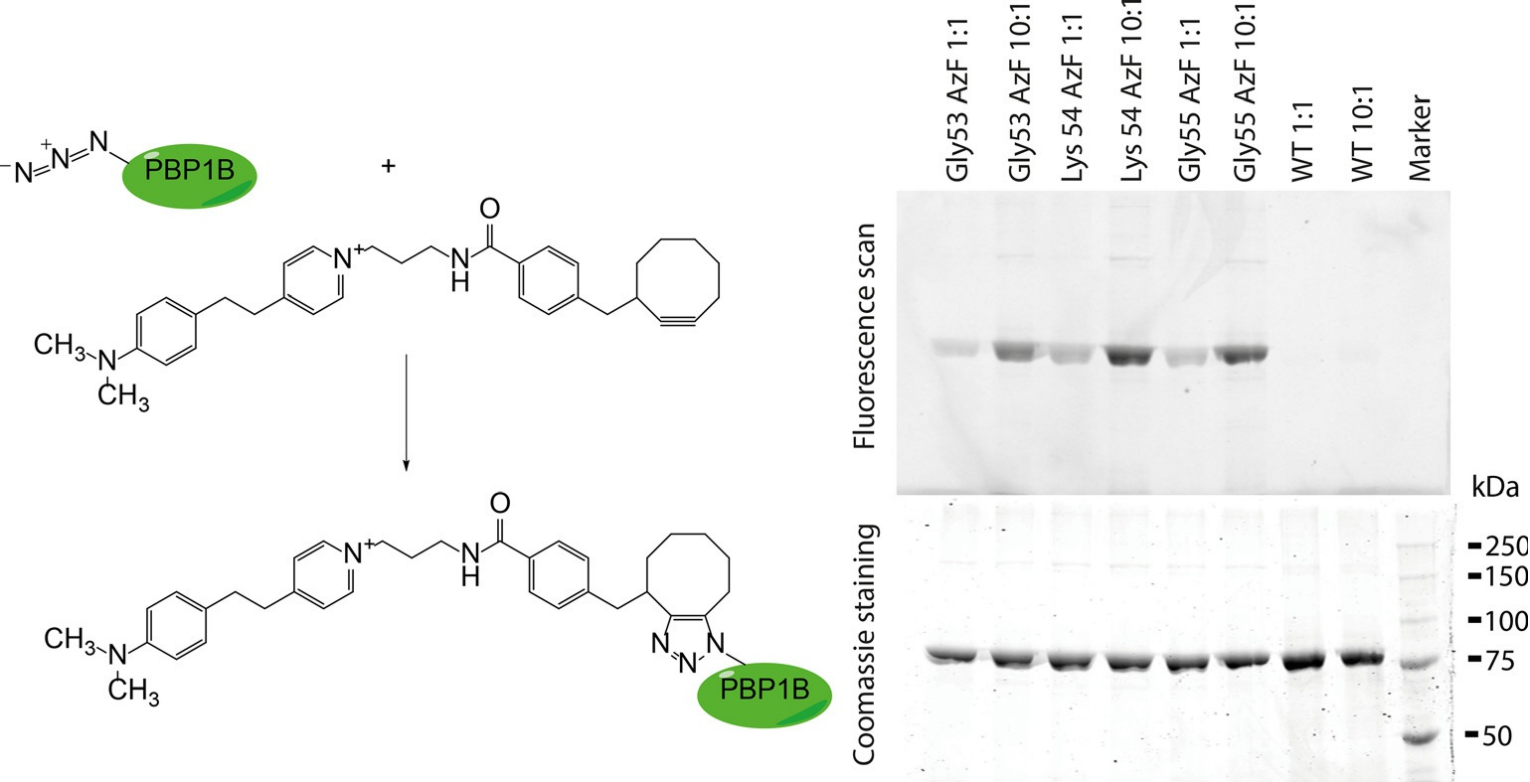
Left: The three PBP1B variants have an incorporated azide that specifically reacts with a cyclooctyne‐containing fluorescent dye. Right: The variants were expressed in *E. coli*, purified by nickel affinity chromatography, and incubated overnight at RT with the cyclooctyne‐containing fluorescent dye Megastokes 608. Samples were separated by SDS‐PAGE. The gel was scanned with a florescence scanner (upper image) to visualize the labeled proteins and then stained with Coomassie blue (lower) to visualize total protein content. WT protein was not labeled. The ratios of dye to protein are indicated (above).

An azide‐containing unnatural amino acid was incorporated in the N‐terminal tail of the protein by using nonsense suppression mutagenesis. This azide was used to covalently attach the protein to the dibenzylcyclooctyne‐coated chip surface by copper‐free click chemistry. Because this is a new method and there is no information about the efficiency of this immobilization method and the dependency on the position of the azide, we substituted three adjacent amino acids in the N‐terminal region of PBP1B for the unnatural amino acid *p*‐azidophenylalanine. By site‐directed mutagenesis, the codon for Gly53, Lys54, or Gly55 of PBP1B was mutated to an amber (TAG) codon. When each mutated PBP1B variant was expressed with an orthogonal tRNA/aminoacyl‐tRNA synthase pair that recognizes TAG and is specific for the incorporation of the unnatural amino acid *p*‐azidophenylalanine, three mutant proteins were produced: azidophenylalanine in place of either Gly53, Lys54, or Gly55. In order to verify azide incorporation, we incubated purified protein with a fluorescent dye containing a cyclooctyne group, which spontaneously reacts with the azide (Figure 2 A). In this way, the azide‐containing proteins are fluorescently tagged. This reaction mixture was separated by SDS‐PAGE, and the gel was scanned with a florescence scanner to visualize labeled protein, then stained with Coomassie Brilliant Blue to assess the total protein content loaded. The azide was indeed incorporated into all three mutant proteins, according to the fluorescence signals (Figure 2 B). An excess of cyclooctyne‐containing dye was needed for an efficient reaction under these conditions (1:1 vs 10:1). Incubation with wild‐type protein did not result in fluorescence, thus showing that the reaction was specific.

### Azide‐containing PBP1B proteins show both GTase and TPase activity in an in vitro peptidoglycan synthesis assay

For the implementation of our SPR method, we used fully active PBP1B proteins (both GTase and TPase activities). We performed an in vitro PG synthesis assay to verify that the azide‐containing proteins retained both activities. Protein was incubated in a buffer containing all the ingredients needed for activity, supplemented with fluorescently labeled lipid II for the detection of the produced polymers. This labeled lipid II cannot be used as a substrate for the crosslink‐forming TPase reaction, as the position of the attached fluorophore is the position used in crosslink formation. Furthermore, *E. coli* PBP1B needs a lipid II version with a *meso*‐diaminopimalic acid at this position of the donor peptide for crosslink formation, and the labeled version originated from a lysine version. Therefore, unlabeled *meso*‐diaminopimalic acid lipid II was included in the mixtures for the TPase reaction.

**Figure 3:**
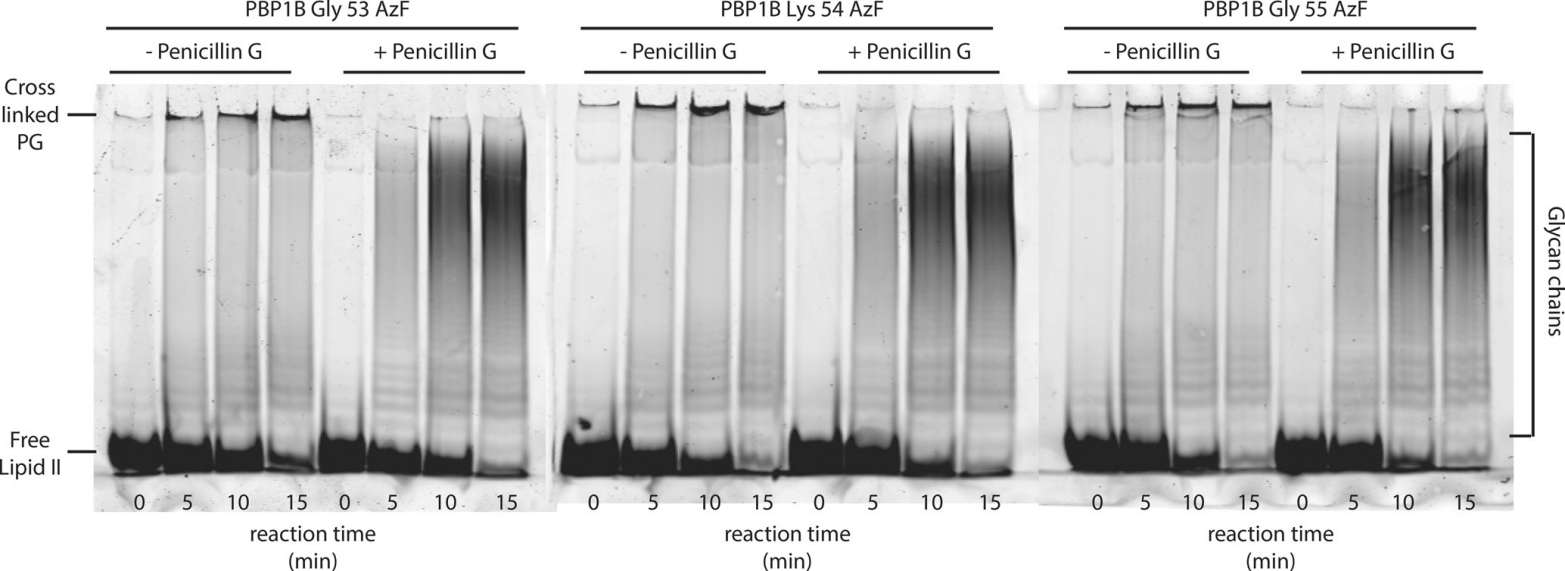
In vitro PG synthesis assay shows that all three azido‐protein variants perform both GTase and TPase reactions. Purified PBP1B was incubated at 30 °C with a mixture of labeled and unlabeled lipid II, and samples were taken at the indicated time‐points. Produced glycan chains were separated by size on a Tris/Tricin SDS‐PAGE gel. The overall increase in chain length and decrease in intensity of the free lipid II band over time indicates GT activity of the three mutant proteins. TPase activity is detected by the high intensity bands at the top of the gel for reactions with penicillin G, which inhibits the TPase reaction. Cross‐linked PG does not enter the gel.

In order to analyze solely GTase activity, penicillin G was added to some of the reaction mixtures (to inhibit TPase activity). As a result of GTase activity, sugar moieties of lipid II were polymerized into glycan strands. These glycan strands were separated by Tris/Tricine SDS‐PAGE.^24^, ^25^, ^26^, ^27^, ^28^, ^29^ The glycan polymers separated by size, with smaller ones visible as separated bands in the lower region of the gel, and longer strands as a smear in the higher region (Figure 3, right). Unincorporated lipid II monomers are visible at the bottom of the gel (Figure 3, bottom left). Crosslinked PG is visible as a band at the bottom of the well in the absence of penicillin G (Figure 3, top left), because the crosslinked PG network is too large to enter the gel. These results show that the three mutant proteins are fully active (both GTase and TPase) in this in vitro PG synthesis assay.

### Use of azide‐incorporated PBP1B for site‐specific immobilization on an SPR chip

*Optimization of immobilization conditions*: For immobilization of the azide‐containing PBP1B variants, we used an amine‐functionalized chip surface to perform the SPR experiments. First, it was functionalized by using the amine‐reactive sulfo‐dibenzylcyclooctyne‐NHS ester. As the efficiencies of functionalization and the subsequent click‐reaction with the azide in the proteins were not known, we varied the concentration of sulfo‐dibenzylcyclooctyne‐NHS ester from 0.25 to 1 mm and the protein concentration from 0.04 to 0.5 μm. In order to identify the optimal conditions for PBP1B immobilization and interaction measurement, we used the well‐characterized interaction between PBP1B and LpoB as a test system, as the kinetic parameters of this interaction have been well established.^18^, ^24^, ^30^

The amount of protein bound to a chip surface is represented by the response of local ligand (RLL) value, expressed in resonance units (RUs). 1 RU corresponds to approximately 1 pg mm^−2^, and the binding capacity (*R*_max_) depends on the amount of protein immobilized on the chip surface according to *R*_max_=(analyte MW/ligand MW)×RL×Sm (stoichiometric ratio). A typical RLL values for our type of measurement is 1000 RU.

**Figure 4:**
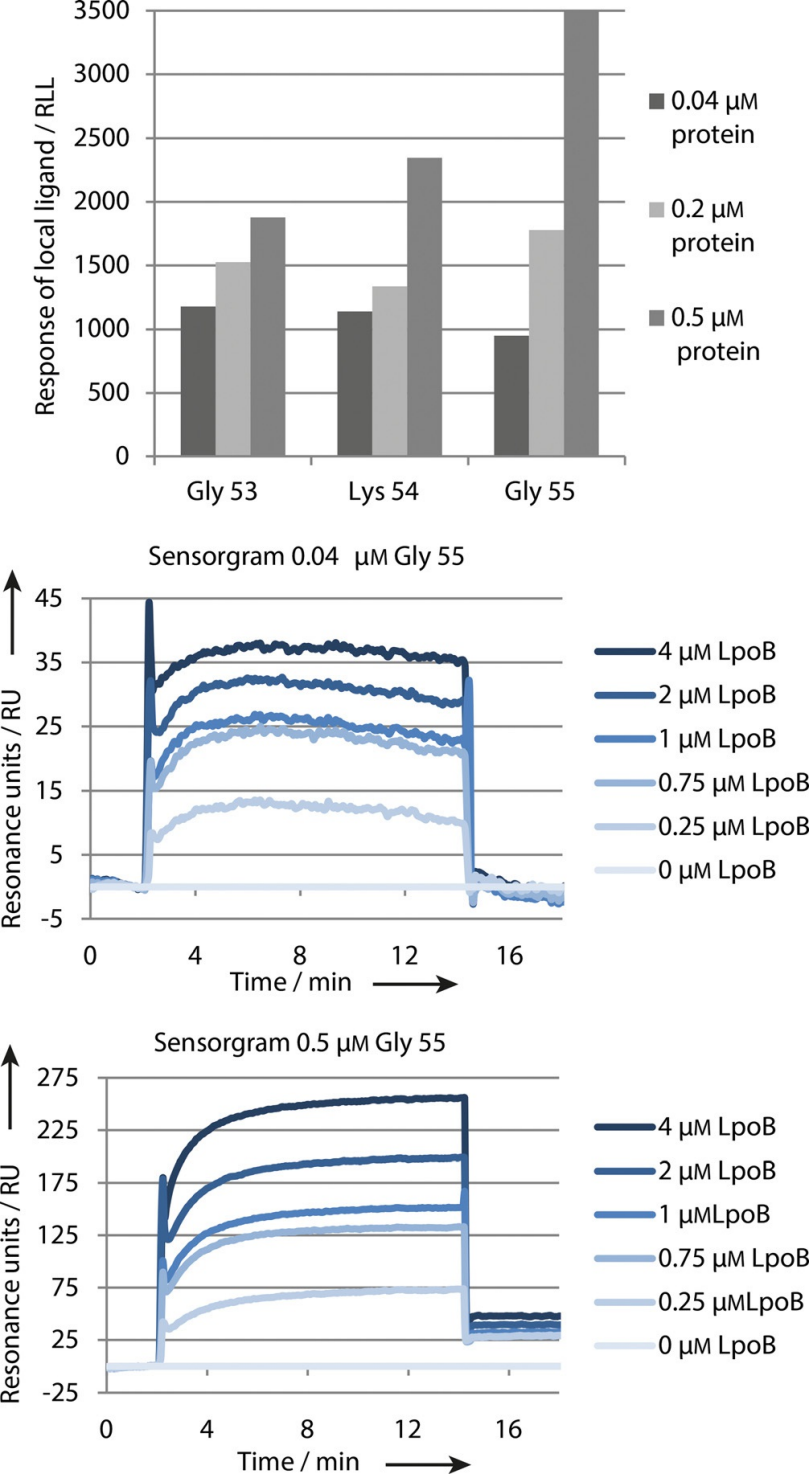
Top: Immobilization of the three azido‐protein variants on the SPR surface. The amount of immobilized protein is represented by the RLL value: all thee variants were immobilized on the SPR chip surface at different protein concentrations. Middle and bottom: Example SPR response curves for two PBP1B Gly55AzF immobilized spots upon LpoB injection.

All three azido‐protein variants were well immobilized on the chip surface, thus suggesting that the position of the azide is not crucial for immobilization efficiency in this case (Figure 4). The highest protein concentration tested (0.5 μm) resulted in the highest amount of immobilized protein on the chip without causing protein aggregation, which would render the protein inactive. Sulfo‐dibenzylcyclooctyne‐NHS ester concentration did not affect immobilization efficiency or the SPR signals (data not shown). Therefore, we used 0.5 μm protein and 1 mm sulfo‐dibenzylcyclooctyne‐NHS ester with variant Gly55 in further experiments. This variant was slightly more active in the in vitro PG synthesis assay than PBP1B‐Gly53, and on average produced SPR curves with a higher signal than PBP1B‐Lys54 upon injection of LpoB (PBP1B‐binding analyte). We decided to include an azidoethanol blocking step because this resulted in slightly higher responses under the above conditions and, more importantly, in order to block possible hydrophobic interactions between injected protein and free cyclooctyne groups on the chip. A blocking step (with ethanolamine) is included in the ampicillin immobilization method, so including a blocking step in our method also allowed a better comparison between the two methods. The results of the all optimization experiment are shown in Table S2 in the Supporting Information.

### Do the immobilized PBP1B variants still interact with LpoB in a similar way?

Next, we immobilized PBP1B on every spot of the chip (except for some control spots) with the optimized conditions. Injection of LpoB over the PBP1B‐immobilized SPR surface resulted as an increase in RU; stopping injection resulted in release of the interacting molecules, and thus a decrease in RU.

**Figure 5:**
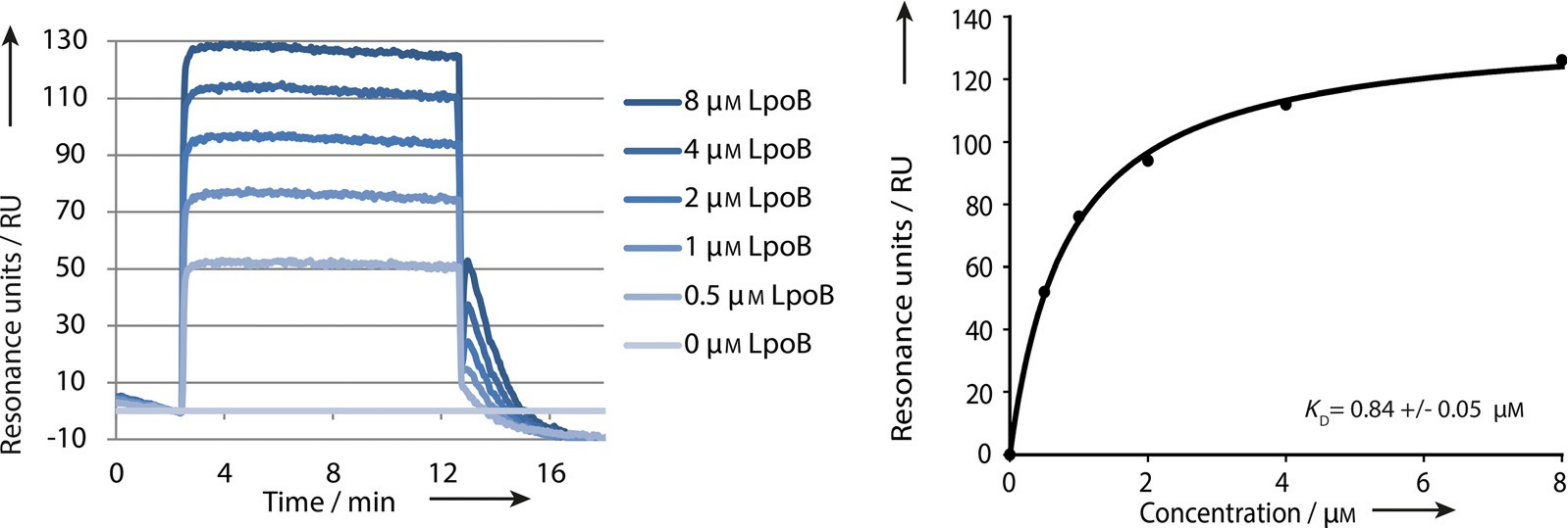
Left: SPR sensorgram for injection of LpoB at different concentrations over a PBP1B immobilized on a chip. Right: Analysis of the SPR data by nonlinear regression for one‐site saturation in the simple ligand‐binding tool of SigmaPlot. The plot of maximum RU against analyte concentration determines the equilibrium constant: *K*_D_=0.84±0.05 μm, which is close to the 0.81±0,08 μm previously found.^18^

The sensorgram for the injection of LpoB over immobilized PBP1B (Figure 5, left) shows that LpoB has a very quick association with PBP1B, as published before.^18^ The immediate rise in RU to the equilibrium made it impossible to determine the association rate constant. The same holds for the dissociation of LpoB from PBP1B when injection ceased. The maximum resonance unit (maxRU) values for the different analyte concentrations were plotted by non‐linear regression with the formula *y*=*B*_max_ *x*/(*K*_D_+*x*) (one site saturation in the simple ligand‐binding tool of SigmaPlot), in order to determine the equilibrium constant. This resulted in calculated *K*_D_ values of 0.71–0.97 μm with a standard deviation of ±0.052, which is close to the 0.81±0.08 μm found by Egan et al.^18^ The small differences in equilibrium constant could have arisen from slight differences in buffer composition, pH or temperature at which the measurements were performed.

These results show that this new PBP1B‐immobilization technique, with an azide incorporated in the protein cytoplasmic tail, is a good alternative to the ampicillin‐immobilization method for SPR experiments. We show that it produces similar results when analyzing the interaction of PBP1B with LpoB. We have not yet identified the specific interactions (of the TPase domain of PBP1B) that would be preferably analyzed by our new immobilization strategy. Incubation of PBP1B with ampicillin prior to immobilization did not alter the binding of LpoB to PBP1B (data not shown), thus suggesting that the interaction is independent on the state of the TPase domain. This also implies that the activation of the TPase by LpoB does not depend on the availability of a TPase substrate, consistent with primary activation of the GTase by LpoB.^18^, ^31^

This new immobilization method can be used for the immobilization of any desired protein, and creates the possibility to control the orientation of the protein by the site specific incorporation of the azide. Replacing different surface amino acids and homogeneously orientating the protein on the chip surface opens the possibility to study the topology‐dependence of interactions of membrane proteins.

## Experimental Section

**Bacterial strains and plasmids**: *Escherichia coli* DH5α cells were used for DNA amplification. *E. coli* BL21(DE3) cells were used for protein expression. Plasmid *p*DML924 carrying the m*rcB* gene, which encodes the N‐terminal His_6_‐tagged variant PBP1Bγ (a gift from Mohammed Terrak, University of Liege, Belgium),^15^ was used for overexpression of PBP1B and as a template for the generation of PBP1B mutants. Plasmid *p*Evol‐pAzF encoding the orthogonal aminoacyl tRNA synthase‐tRNA_CUA_ pair was used for incorporation of *p*‐azidophenylalanine at the site of an amber mutation. Plasmid pET28LpoB (signal sequence and lipid anchor of LpoB replaced by an oligohistidine tag, LpoB(sol)) was used for the overexpression of LpoB (sol).^18^

**Site‐directed mutagenesis**: The amber mutants were created by mutagenesis PCR (primers in Table S1). The reaction mixture contained fwd and rev primer (125 ng), dNTPs (10 mm each, 1 μL), template DNA (DNA at an end concentration of 1.23 ng μL^−1^, 1 μL of a 61.5 ng μL^−1^) and Phusion DNA polymerase (1 U, 0.5 μL; Thermo Fisher Scientific) in a total volume of 50 μL in [1× Phusion buffer]. We performed 17 cycles of 30 s at 98 °C, 1 min at *T*_m_ (depending on the primers), and 5 min at 72 °C. PCR products were digested with DpnI (10 U; Fermentas) and amplified in *E. coli* DH5α. Sequencing confirmed the intended mutations.

**Expression and purification of azide‐containing PBP1B and LpoB**: *E. coli* BL21 (DE3) cells were co‐transformed with pDML924 containing the amber mutation and *p*Evol‐pAzF, and grown at 37 °C to OD_600_=0.5–0.6. tRNA/tRNA synthase production was induced with arabinose (0.04 %), and freshly prepared *p*‐azidophenylalanine, (0.1 mm in 1 m NaOH) was added. After 30 min, protein production was induced with IPTG (1 mm). After 2 h, cells (1 L culture) were harvested by centrifugation, and resuspended in buffer A (18 mL; Tris**⋅**HCl (20 mm pH 8.0), NaCl (300 mm), imidazole (5 mm)) supplemented with PMSF (0.1 mm) and 1 cOmplete EDTA‐free protease inhibitor tablet (Roche). Cells were lysed by sonification in Sonifier 250 (Branson Ultrasonics, Danbury, CT) with a microtip. Intact cells were removed by centrifugation (3500 *g*, 10 min), then the lysate was centrifuged (200 000 *g*, 90 min, 4 °C) in a WX80 Ultra with a T865 rotor (Sorvall). The membrane fraction (pellet) was solubilized in buffer A (12 mL) supplemented with Triton X‐100 (2 %), by stirring for 2 h at 4 °C. Insoluble material was removed by centrifugation (200 000 *g*, 90 min, 4 °C). Solubilized proteins were incubated with Ni^‐^Sepharose beads (300 μL; GE Healthcare) overnight at 4 °C. The beads were centrifuged (3500 *g*, 3 min, 4 °C), and the unbound fraction was discarded. The beads were washed with buffer A (5×10 mL) containing imidazole (50 mm) and Triton X‐100 (0.1 %), then with buffer A (3×2 mL) containing imidazole (100 mm) and Triton X‐100 (0.1 %). Proteins were eluted in buffer A (4×2 mL) containing imidazole (500 mm) and Triton X‐100 (0.1 %). Fractions were dialyzed by using a 500 Da membrane against dialysis buffer A (Tris**⋅**HCl (20 mm pH 8.0), NaCl (300 mm), MgCl_2_ (10 mm), Triton X‐100 (0.1 %), glycerol (10 %)) at 4 °C for 48 h. Protein content for all three mutants was analyzed with a BSA range on a Coomassie‐stained gel (±0.2 μg μL^−1^, =±2.25 μm). Protein was stored at −20 °C.

*E. coli* BL21 (DE3) cells harboring plasmid pET28LpoB were grown at 30 °C to OD_578_=0.5–0.6. Overexpression was induced by the addition of IPTG (1 mm), and cells were incubated for a further 3 h at 30° C. Cells were harvested by centrifugation and resuspended in buffer B (Tris**⋅**HCl (25 mm, pH 7.5), MgCl_2_ (10 mm), NaCl (500 mm), imidazole (20 mm), glycerol (10 %)), supplemented with DNase (Sigma bovine pancreatic DNase, 1 spatula point), protease inhibitor cocktail (Sigma 1/1000 dilution), and PMSF (0.1 mm). Cells were disrupted by sonication. The lysate was centrifuged (130 000 *g*, 1 h, 4 C), and the supernatant was applied to a 5 mL HisTrap HP column (GE Healthcare) attached to an ÄKTA Prime+ system (GE Healthcare;1 mL min^−1^). The column was washed with four volumes of buffer B, followed by elution of bound proteins with buffer C (Tris**⋅**HCl (25 mm, pH 7.5), MgCl_2_ (10 mm), NaCl (500 mm), imidazole (400 mm), glycerol (10 %)). In order to remove the His tag from His‐LpoB(sol), restriction grade thrombin (50 U mL^−1^; Merck Millipore) was added. The protein was dialyzed against dialysis buffer B (Tris**⋅**HCl (25 mm, pH 8.3), NaCl (100 mm), glycerol (10 %)) for 18 h at 4 °C. LpoB(sol) was applied to a 5 mL HiTrap Q HP column (GE healthcare) attached to an ÄKTA Prime+ (GE Healthcare) at 0.5 mL min^−1^ and collected in the flow‐through. LpoB(sol) was concentrated to 4–5 mL in a VivaSpin column (MW cut‐off 6000 Da) and applied to a Superdex 200 HiLoad 16/600 column at 1 mL min^−1^ for size‐exclusion chromatography in buffer D (HEPES/NaOH (25 mm, pH 7.5), NaCl (1 m), glycerol (10 %)). Finally, the protein was dialyzed against storage buffer (HEPES/NaOH (25 mm, pH 7.5), NaCl (500 mm), glycerol (10 %)) and stored at −80 °C.^18^

**Confirmation of the presence of the azide in PBP1B mutants by coupling of cyclooctyne‐containing fluorescent dye and SDS‐PAGE analysis**: Protein solution (10 μL, 2.25 μm) was incubated with MegaStokes dye 608 (2.25 or 22.5 μm; Sigma–Aldrich) overnight at RT. Laemmli sample buffer (without DTT) was added, and samples were run on an 8 % SDS‐PAGE gel. Fluorescence was analyzed with a Typhoon 9400 scanner (GE healthcare), and protein content were estimated by Coomassie Blue staining.

**Protein activity test using an in vitro PG synthesis assay and visualization on Tris/Tricin SDS‐PAGE**: PBP1B (1 μm) was incubated with ATTO550 lipid II (10 μm; synthesized as previously described^32^, ^33^) and *m*‐DAP lipid II (100 μm), in HEPES (20 mm, pH7.5) with NaCl (150 mm), MgCl_2_ (10 mm), and Triton X‐100 (0.05 %) at 30 °C. Penicillin G (1 μg) was added to some reactions to be able to analyze only the GT reaction. Samples (15 μL) were taken at various time‐points, and the protein was inactivated by boiling (99 °C, 5 min). The samples were dried in a SpeedVac, dissolved in sample buffer (4 μL; Tris**⋅**HCl (60 mm, pH 8.8), glycerol (25 %), SDS (2 %)), and analyzed by Tris/Tricine SDS‐PAGE. Gels were prepared at a final concentration of 9 % T, 2.6 % C (T: total percentage of both acrylamide and bisacylamide; C: percentage of bisacrylamide relative to T). This was prepared in gel buffer (Tris (0.5 m, pH 8.45), SDS (0.13 %)). Gels were run with anode buffer (Tris (0.1 m, pH 8.8)) and cathode buffer (Tris (0.1 m, pH 8.25), tricine (0.1 m), SDS (0.1 %)) at 30 mA (maximum voltage 200 V). Gels were scanned in the Typhoon 9400.

**SPR studies**: An IBIS‐MX96 (IBIS Technologies, Enschede, The Netherlands) was used. PBP1B variants were immobilized on the surface of a SensEye P‐NH2 sensor (IBIS) coated with sulfo‐dibenzylcyclooctyne‐NHS ester (Jena Bioscience, Jena, Germany). After activation of the chip, the spots were coated for 60 min with sulfo‐dibenzylcyclooctyne‐NHS ester (1, 0.5, or 0.25 mm in HEPES (20 mm, pH 7.5)). After a rinse with PBS, PBP1B (0.5, 0.2, or 0.04 μm) in running buffer (Tris/maleate (10 mm, pH 7.5), NaCl (150 mm), Triton X 100 (0.05 %)) was spotted for 30 min. After a rinse with running buffer, excess sulfo‐dibenzylcyclooctyne‐NHS ester was blocked with azidoethanol (0.5 m in running buffer) for 10 min. The amount of immobilized protein ranged from 1000 to 3000 RU (1 RU corresponds to approximately 1 pg of protein per mm^2^). Some spots were controls with different treatments (Table S2). Analytes in running buffer were injected at different concentrations: 2 min baseline, 10 min association, 5 min dissociation, and two times 30 s of regeneration with running buffer (containing NaCl (1 m)) were recorded. The sensorgrams were evaluated with SPRintX (IBIS), and the parameters of the interaction were calculated in SigmaPlot (Systat Software, San Jose, CA) by using the simple ligand‐binding tool and one‐site saturation.

## Supporting information

As a service to our authors and readers, this journal provides supporting information supplied by the authors. Such materials are peer reviewed and may be re‐organized for online delivery, but are not copy‐edited or typeset. Technical support issues arising from supporting information (other than missing files) should be addressed to the authors.

Supplementary
