# Supplementary material for: Site‐Specific Immobilization of the Peptidoglycan Synthase PBP1B on a Surface Plasmon Resonance Chip Surface
Source: Chembiochem. 2016 Nov 7;17(23):2250–6. doi: 10.1002/cbic.201600461 (PMC5298014; doi:10.1002/cbic.201600461)
Supplement: Supplementary file 1 — Supplementary [file CBIC-17-2250-s001.pdf]

CHEMBIOCHEM

Supporting Information

Site-Specific Immobilization of the Peptidoglycan Synthase PBP1B on a Surface Plasmon Resonance Chip Surface

Inge L. van't Veer,[a] Nadia O. L. Leloup,[b] Alexander J. F. Egan,[c] Bert J. C. Janssen,[b] Nathaniel I. Martin,[d] Waldemar Vollmer,[c] and Eefjan Breukink*[a]

cbic_201600461_sm_miscellaneous_information.pdf

Table S1

(G) gly 53	fwd<br>rev	GGTAAGGGCAAATAGAAAGGGCGTAAGC<br>GCTTACGCCCTTTCTATTTGCCCTTACC
(K) lys 54	fwd<br>rev	GGTAAGGGCAAAGGCTAGGGGCGTAAGC<br>GCTTACGCCCCTAGCCTTTGCCCTTACC
(G) gly 55	fwd<br>rev	GGCAAAGGCAAATAGCGTAAGC<br>GCTTACGCTATTTGCCTTTGCC

Table S2

Spot	Protein	Coating	RLL	max RU	kD (µM)
23	0,04 µM gly53	0,25 mM	1401	70	0,452
24	0,04 µM gly53	0,5 mM	1098	45	0,535
2	0,04 µM gly53	1 mM	1387	54	1,374
7	0,04 µM gly53	1 mM	1175	61	0,953
20	0,2 µM gly53	0,25 mM	1798	53	0,689
21	0,2 µM gly53	0,5 mM	1725	54	0,798
22	0,2 µM gly53	1 mM	1367	59	0,597
10	0,2 µM gly53	1 mM	1526	46	0,803
17	0,5 µM gly53	0,25 mM	1929	37	0,565
18	0,5 µM gly53	0,5 mM	2618	68	0,849
19	0,5 µM gly53	1 mM	2413	87	0,781
3	0,5 µM gly53	1 mM	1878	164	1,123

Spot	Protein	Coating	RLL	max RU	kD (µM)
4	0,04 µM lys54	0,25 mM	363	-	-
6	0,04 µM lys54	0,5 mM	1041	62	1,293
8	0,04 µM lys54	1 mM	657	38	1,068
14	0,04 µM lys54	1 mM	1138	31	1,594
28	0,2 µM lys54	0,25 mM	1094	34	0,515
29	0,2 µM lys54	0,5 mM	1872	61	0,655
30	0,2 µM lys54	1 mM	1976	81	0,727
16	0,2 µM lys54	1 mM	1335	68	2,449
25	0,5 µM lys54	0,25 mM	1971	44	0,492
26	0,5 µM lys54	0,5 mM	1323	44	0,387
27	0,5 µM lys54	1 mM	2408	106	0,723
5	0,5 µM lys54	1 mM	2345	180	1,044

Spot	Protein	Coating	RLL	max RU	kD (µM)
31	0,04 µM gly55	0,25 mM	1399	56	0,414
32	0,04 µM gly55	0,5 mM	1219	51	0,517
1	0,04 µM gly55	1 mM	48	-	-
11	0,04 µM gly55	1 mM	949	35	0,797
36	0,2µ M gly55	0,25 mM	995	32	0,578
37	0,2 µM gly55	0,5 mM	1747	80	0,767
38	0,2 µM gly55	1 mM	1883	108	1,071
48	0,2 µM gly55	1 mM	1777	78	1,117
33	0,5 µM gly55	0,25 mM	3271	132	0,679
34	0,5 µM gly55	0,5 mM	2251	115	0,66
35	0,5 µM gly55	1 mM	2155	109	0,733
47	0,5 µM gly55	1 mM	3522	253	0,992

Spot	Protein	Coating	RLL	max RU	kD (µM)
39	0	0	32		
9	0	0	45		
40	0	0	14		
12	0	0	-172		
13	0	0	-179		
15	0	0	-247		
41	0	0,25 mM	317		
42	0	0,5 mM	751		
43	0	1 mM	868		
44	0	0,25 mM	721		
45	0	0,5 mM	746		
46	0	1 mM	962		
